# Patient online access to general practice medical records: A qualitative study on patients’ needs and expectations

**DOI:** 10.1177/18333583221144666

**Published:** 2023-01-19

**Authors:** Rosa RLC Thielmann, Ciska Hoving, Esther Schutgens-Kok, Jochen WL Cals, Rik Crutzen

**Affiliations:** 1 Department of Health Promotion, Care and Public Health Research Institute, 5211 Maastricht University, Maastricht, The Netherlands; 2 Department of Family Medicine, Care and Public Health Research Institute, 5211 Maastricht University, Maastricht, The Netherlands

**Keywords:** electronic health records, health records, personal, medical records, patient access to records, patient portals, patient participation, qualitative research, health information management

## Abstract

**Background:**

Patient online access to medical records is assumed to foster patient empowerment and advance patient-centred healthcare. Since July 2020, patients in the Netherlands have been legally entitled to electronically access their medical record in general practice. Experience from pioneering countries has shown that despite high patient interest, user rates often remain low. How to best support implementation depends on individual needs and expectations of patient populations, which are as yet unknown in the Dutch context.

**Objective:**

To understand Dutch patients’ needs and expectations with regard to online access to their medical record in general practice.

**Method:**

Twenty participants completed semi-structured individual interviews via video or telephone call. Transcripts of interviews underwent template analysis combining deductive and inductive coding using Atlas.ti software.

**Results:**

Patients’ needs and expectations ranged across three overlapping areas: (i) prerequisites for getting online access; (ii) using online access; and (iii) the impact on interaction with healthcare providers. Patients expected benefits from online access such as better overview, empowerment and improved communication with their general practitioner but identified needs regarding technological difficulties, data privacy and complex medical language in their record.

**Conclusion:**

The concerns and obstacles participants identified point towards the need for organisational changes in general practice, for example, adjusted documentation practices, and the key role of the general practitioner and staff in promoting and facilitating online access.

**Implications:**

Implementation strategies addressing needs identified in this study may help to unlock the full potential of online access to achieve desired outcomes of patient involvement and satisfaction.

## Introduction

Patient empowerment and patient-centeredness are increasingly regarded as critical quality attributes in healthcare (Wolfe, 2001). While there is no widely accepted definition of those terms, conceptualisations often illustrate an ideological shift from paternalistic to increasingly participation-based healthcare in which communication and respecting the patients’ voice become key values (Castro et al., 2016). A promising mechanism to enable patient participation in healthcare is patients’ online access (hereafter “online access”) to medical records (Irizarry et al., 2015). Online access to medical records was ascribed the potential to facilitate patient informed decision-making (Irizarry et al., 2015) by improving the patients’ health knowledge (Han et al., 2019). Informed decision-making is the process resulting in decisions that the patient makes based on relevant and good quality knowledge that reflect the patients’ values and that are behaviourally implemented (Marteau et al., 2001; Bekker et al., 1999). Involving patients more in decisions about their health has the potential to improve affective-cognitive patient outcomes such as patient satisfaction (Shay and Lafata, 2014).

Recently, in July 2020, patients in the Netherlands became legally entitled to access parts of their medical record electronically in primary care (mainly care delivered through the general practitioner [GP]). Access is mainly facilitated via online patient portals which are directly tethered to the medical record held by the GP. Access is safeguarded by two-factor authentication. In patient portals, patients can view medication and allergy lists, medical notes, and diagnostic test results (HealthIT, 2019). Patients’ demand can be assumed high, as 88% of the adult Dutch population finds it important to have online access to their medical data (Netherlands Patients Federation, 2020). However, experiences from pioneering U.S. settings show user rates of only 15–30% despite high patient interest (Lyles et al., 2020), as well as long lists of challenges in the adoption of online access for both patient and healthcare provider. These challenges are attributable to technological aspects, factors related to the medical practice and provider and characteristics and according needs of the patient (Niazkhani et al., 2020).

Research on patient needs in various countries among diverse patient populations point towards the following barriers in the use of online access: lack of awareness and insufficient training or instructions regarding use of online access (Powell, 2017), a complex and complicated interface of the online environment (McGinn et al., 2011; Ose et al., 2017), privacy and security concerns (McGinn et al., 2011; Powell, 2017) and anticipated distress and anxiety when receiving sensitive or incomprehensible medical information through technology without the presence of a healthcare provider (Baun et al., 2020; Jilka et al., 2015). Further, facilitators for the use of online access include encouragement from the healthcare provider (Powell, 2017), expecting benefits from use (Crameri et al., 2020; McGinn et al., 2011) – especially the benefit of improved communication and relationship with the provider (Ose et al., 2017; Powell, 2017) – and the feeling of empowerment and enhanced control (Crameri et al., 2020; Powell, 2017). Further, previous findings indicate that patients with certain characteristics experience more difficulties in using online access than others. Older adults (Logue and Effken, 2012; Lyles et al., 2020), patients with limited health and digital literacy (Emani et al., 2012; Lyles et al., 2020), low socioeconomic status (Emani et al., 2012; Roblin et al., 2009; Yamin et al., 2011) and members of ethnic minorities (Roblin et al., 2009; Yamin et al., 2011) are more prone to face challenges in accessing and meaningfully engaging with their medical data.

Previous research has consistently pointed towards the importance of understanding patients’ needs, expectations and perspectives to unfold the full potential of online access (Crameri et al., 2020; Fragidis and Chatzoglou, 2018; McGinn et al., 2011; Entzeridou et al., 2018). Moreover, best practice seems to depend on the type and level of access (e.g. opt-in/opt-out) (Nøhr et al., 2017) and differs between patient populations in various geographic locations, sociocultural contexts and stages of online access implementation (Prey et al., 2016). The recent introduction of online access in general practice in the Netherlands provided the opportunity to explore patients’ needs and expectations in this early stage of implementation to subsequently integrate these in the development and improvement of strategies that support the implementation of online access. To this end, this study aimed to identify needs and expectations of Dutch patients in regard to online access to their medical record in general practice.

## Method

A qualitative study was conducted to reach our study aim. This study is reported in accordance with the Consolidated Criteria for Reporting Qualitative (COREQ) Research Guidelines (Tong et al., 2007).

### Research design

A series of semi-structured individual interviews were conducted. Individual interviews were chosen as a broader range of information may be generated than through focus groups (Guest et al., 2017). An interview guide was developed based on the concepts described in the introduction and on the researchers’ experience in similar fields. The interview guide, patient information letter, informed consent form and demographic questionnaire can be found at the Open Science Framework (https://osf.io/8ezyu/).

### Participants and recruitment

Eligible for participation were adults (18+) from the Dutch population who had been in contact with their GP at least once in the six months prior to recruitment. For recruitment, printed flyers were randomly distributed via letterboxes in a mid-sized city in the southern part of the Netherlands, a digital flyer was uploaded on various social media platforms, and one researcher (ESK, not conducting interviews) recruited participants from her network via personal invite. Interested individuals could either contact the main researcher (RT) to ask questions or directly access a digital form with detailed information about the study and request for informed consent. The form also included a five-item background questionnaire about sociodemographic characteristics that earlier research showed to influence use of online access to medical data: age (Lyles et al., 2020), gender identity (Miles et al., 2016), cultural background, highest level of education completed (Emani et al., 2012) and having a chronic disease (Niazkhani et al., 2020). Based on the answers, purposeful sampling was employed to achieve a heterogenic sample. Fifty-one people showed interest in participation by filling in the digital form.

### Data collection

As data were collected while contact-limiting COVID-19 measures were in place, all participants preferred to be interviewed via videocall or telephone instead of in person (although this option was provided as well). Interviews were conducted in Dutch. After a first inquiry of participants’ experience with online access, they were shown a 40 second videoclip explaining online access to assure understanding of the interview topic. This was deemed necessary as participants were expected to have little experience with online access due to the recent introduction and low user rates (OPEN-Eerstelijn, 2020). Participants were asked questions about 1) their previous experiences with online access to their GP medical record; 2) whether, under which circumstances, and how they (would) make use of it; 3) expected or experienced advantages and disadvantages, specifically the impact on the GP-patient relationship; and 4) what they perceived as barriers and facilitators for use and consequent needs. Other topics participants mentioned were explored as well. Duration of interviews was between 30 and 55 minutes. After the interview, participants were reimbursed with a 20-euro gift voucher. Audio was recorded with *QuickTime Player* and fieldnotes were taken. Interviews took place between February and May 2021 until data saturation was reached (i.e. three consecutive interviews did not produce new knowledge relevant to the research question) (Mason, 2010). The audio recordings were transcribed verbatim. Transcripts were returned to participants to confirm accuracy and approve further use.

### Data analysis

Sociodemographic data from the questionnaire were summarised with Microsoft *Excel*. The coding software *Atlas.ti 9* was used for the analysis of transcripts. Due to its structured yet flexible approach, the steps of template analysis (Brooks et al., 2015), a form of thematic analysis (Braun and Clarke, 2006), were followed: The coders (RT and ESK) (i) familiarised themselves with the data, (ii) in an iterative process carried out preliminary coding on a sub-set of the data (25%) with both tentative a priori as well as inductive themes, (iii) organised themes into clusters, (iv) defined an initial coding template, (v) applied the initial template to 10% of the data, discussed coding and modified the template as necessary, and (vi) applied this version of the template to another 10% of the dataset based on which inter-coder-agreement was calculated. Krippendorff’s alpha (α) was used because of its advantages compared to other common measures (Hayes and Krippendorff, 2007). As α was 0.82, the template could be considered as reliable (Krippendorff, 2004) and was applied to the full dataset by RT.

### Ethics approval

The study was approved by the Maastricht University Faculty Research Ethics Committee (approval number: FHML-REC/2020/119). Digital and verbal informed consent was obtained before the start of each interview. Participant data were treated confidentially and the possibility to trace transcripts back to specific participants was removed after participants’ check for transcript accuracy.

## Results

After interviewing 20 participants, data saturation was reached. Purposeful sampling resulted in a group of 12 women and 8 men. Six participants were aged 18–34, four were aged 35–49, six were aged 50–65, and four were older than 65 years. Twelve participants completed higher or university education. Five participants reported being diagnosed with a chronic disease. Two participants had a cultural background different from Dutch. Four participants had used online access prior to the interview. Most participants considered online access as “useful” and a “good development”. For many, online access matched their feeling of being entitled to access their own data. Some had mixed feelings and were unsure yet whether they would want online access, and a few stated they see no use or prefer to not be more involved in their healthcare. Overall, participants found it important that their individual choice was respected.

During template analysis, needs and expectations in regard to online access were grouped into three overlapping areas: (i) prerequisites for getting online access, (ii) using online access, and (iii) impact on interaction with healthcare providers.

### Prerequisites for getting online access

Prerequisites necessary for participants to get online access pertained to provision of information, technological conditions and instrumental support. Most participants had not heard about online access before participating in this study. They stated that if they had known about this possibility, they would have asked their GP about it. Many participants seemed indignant that this option was not communicated to them before and planned to get online access in the future.

I didn’t know anything at all. I didn’t know they were working on that. I heard that from [name daughter]. I thought to myself, oh that’s nice. I went right to work on it. But, no, I think most people don’t even know about it. (P9, male, 35–49 years)

Participants saw the GP practice as responsible to inform them about online access. Many wished to be notified either personally or via email. Some imagined it beneficial to see online access advertised in the practice, for example, in the form of a poster or videoclip in the waiting room. “I think they [GPs] should be proactive. Because there are people who indeed don’t know that [the option of online access] and never find out if the doctor doesn’t let them know” (P20, male, 65+).

Most participants identified the degree of complexity for accessing their medical record as substantially influencing their decision and ability to use online access. Many feared difficulties related to technology, specifically a high number of complex actions to create an online access account. Most participants said that they would not want to spend more than 10 minutes to create an account: “Suppose there is one of these double verifications, so then you have to first type a code from your mobile back into your laptop or whatever, yes, then it already gets complicated very quickly” (P3, male, 18–34 years); and “I need to create something that will allow me to get in there, but this creating [of an account], I can’t get it done. I don’t understand these Internet terms there sometimes, so then I stop” (P10, female, 65+).

Participants who already used online access reported mixed experiences: two participants found creating an account easy and intuitive, while two others found it difficult and complicated, and once succeeding also had problems logging back in. Consequently, participants wished for instrumental support in form of clear instructions on how to create an account. A few participants aged 65+ expected to need help from family members or friends who were more experienced with technology. However, the majority found it sufficient but necessary to receive instructions from the GP practice on its website, in an email, pamphlet, or videoclip. Most participants were confident that such instructions would enable them to access their medical record: “They really don’t need to start looking over my shoulder while I’m at the computer trying to create it. But, yes, do provide instruction, on how to create such an account” (P16, female, 50–65 years); and “I actually have to have [instructions] on paper: You have to type that in, there” (P10, female, 65+). However, a few patients also valued a multi-step registration process as double verification would increase protection of their data.

Almost all participants were concerned about the safety and privacy of their medical or personal data when using online access. Mostly, they were afraid that systems get hacked and strangers will have access to their data. Many participants expressed the wish for support from their GP practice in accessing their medical record safely: “The only major drawback is security and privacy. […] I do think that it is more openly accessible if you make it accessible to patients than the way it is organised now”. (P17, female, 50–65); and “I think that they [GP practice] should support you in choosing a good password, or for example a confirmation code by phone or email, so that there is at least some security” (P8, female, 18–35). In contrast, a few participants did not see this as problematic, explaining that they did not perceive their medical record as valuable to anyone else: “Look, my medical records, they are not worth the effort. There is not so much going on there fortunately” (P7, male, 65+ years).

Frequently participants mentioned that they do not want insurance companies or healthcare providers other than their GP to access their data. A few participants believed that their own access would automatically allow access for healthcare providers other than their GP as well. Many expressed concern that disclosure of medical data to third parties would become normal in the future: “If the standard is, in society, that you yourself can see the data, maybe it can also happen at some point that it becomes so normal that data will be further disseminated, so to speak” (P3, male, 18–34 years).

Beyond support for making an account, participants wanted to have realistic expectations conveyed about which data they can access and which role it can have for their healthcare.

Maybe send people a video, for example, […] what is this online environment, or what can it offer you? Well, for example, more insight, that you can prepare well for a conversation. Yes, things like that. And what it cannot offer you, or what it is not. (P8, female, 18–35)

### Using online access

Participants explained how they (intended to) use online access, interact with their medical record, and which immediate benefits and difficulties they expected. Frequently participants mentioned that they expected online access to reduce the cognitive burden to remember all their health information. They mentioned favourably the possibility of re-accessing information that was given during a consultation and reading about their medical history: “How many times have we had it where you’ve had something, and then you come home, and you actually don’t know half of what they [doctors] actually said in there?” (P13, male, 35–49)

Most participants thought that using online access would give them a better overview of their healthcare, especially regarding experienced symptoms, past illness episodes, dates they consulted their GP and when their next medical check-up has to be scheduled: “You really get a better overall picture of, okay, what’s going on here now?” (P3, male, 18–34 years);

As soon as you go into a kind of longer trajectory, then I think it can be of added value to really keep that overview, then also for yourself, like, okay, where are we at the moment, so to speak, what does the GP already know, what are the options, for example, and have I suffered from it before. Those kinds of things (P3, male, 18–34).

Many participants mentioned they want to use online access to detect mistakes in their medical file and valued the opportunity to contribute to rectification. Further, they expected that convincing themselves of the congruence between information given during a consultation and written in their medical record would increase their trust in the GP. Conversely, a few participants also mentioned that earlier experiences of detecting mistakes in their files disrupted their trust in the healthcare provider or caused them distress: “Then of course you can look at it yourself, like: Do I think that’s right, or is something missing?” (P17, female, 50–65 years);

I did see a number of times that the information in my file was actually incorrect. You can say: yes, that’s positive, and then there comes a kind of improvement, that you say to the hospital or GP: I think it’s not correct, you have to correct it. But the trust that you then have in the health care institution, that does not increase, of course. (P1, female, 35–49 years)

They further expected online access would increase their understanding of their health issues. Additionally, many mentioned online access would prompt them to research information relevant to their healthcare on the Internet and thereby expand their health knowledge further: “You see the medical terms, quickly look it up, so that you think: ah, yes, now I understand what he [GP] is talking about” (P3, male, 18–34 years). However, most participants also acknowledged that they would have difficulties to understand and interpret parts of their medical record as they lack medical expertise. Several participants feared that looking for additional explanation on the Internet could lead them to draw the wrong conclusions or find unsettling information: “I think you probably can’t read that yourself” (P6, male, 18–34 years); and “I would go and look it up, and I wonder if that would be right, because then you could also misinterpret it” (P18, female, 18–35).

Therefore, participants emphasised the need for an adapted, simplified language in their medical record which can be understood without having a medical background: “Of course, they [GPs] do have to write it down so that it's clear to a layperson” (P9 male, 35–49 years). Moreover, many participants found it important that the GP communicated sensitive information first face-to-face before making it accessible online for the patient. Additionally, several participants were concerned that using online access could cause them emotional distress when reading unexpected or derogatory remarks from their GP which normally would not be intended for the patient to read. For example, to protect herself from negative feelings, a participant who already used online access decided against reading her medical history:

I don't want to just come across things, for example, imagine you went there with a problem, and you're emotionally sharing your problem. Imagine in the file you read something like “she's overreacting”, or something. […] In that case, if I were to read something back, I wouldn't feel good. (P4, female, 50–65 years)

### Impact on interaction with healthcare providers

Participants expected their use of online access to impact the interaction with their GP and other healthcare providers. They valued the transparency resulting from online access, leading to increased ability to “think along” with their GP, discuss more or be more involved in decisions. They expected that they would feel more equal to their GP in conversations, empowered and less helpless and vulnerable.

It’s easier for you to have a say. About your treatment. About your results. […] Before, of course, it was like this: the doctor said A, and the patient also said A, and then the doctor said no, I mean B, and then the patient also said B. Yes, that's not the case, you become more articulate, and you become more aware of, yes, what do I think, and what do I know, and that's just important. (P2, female, 50–65 years)

Many participants expected that by using online access they would feel better prepared for consultations, enabling them to participate more in the conversation and ask more specific questions. Most participants expected the communication to improve through their increased participation: “The mutual understanding [is] greater. […] If you have to go [to the GP], you are much better consulted, much better prepared. You go there more empowered. I think that's a big advantage” (P2, female, 50–65 years); and “More and more two-way communication, I think. You can ask more targeted questions” (P16, female, 50–65 years).

Further, participants expected that online access would improve the transferal of information to specialists they get referred to by their GP. Many had the feeling that the information transfer from the GP to a specialist was frequently incomplete or absent. Online access would enable them as patients to, either verbally or written, share information about their medical situation themselves.

Normally you say, well, “I don’t have any pills or anything”, or “I don’t know what it's called”. But if you just have it [written] somewhere, then you can say, well, “this is it”. Yeah, I think that is actually good. (P1, female, 35–49 years)

The majority of patients expected fewer telephone conversations with their GP office when having online access, as they would be able to access information, mainly test results, on their own for which they previously required the help of the GP practice.

Usually if I want to see the blood result, for example, I have to call there first, and then the assistant has to go look it up. And now I can click on it myself. Then I see the result already in there. (P13, male, 35–49 years)

However, the majority of participants expected no change in the number of consultations, as they found it important to consult the GP when experiencing a health problem. Only a few participants expected a decrease in the number of consultations as in case of a recurring health issue, they could compare their current symptoms with those in earlier records and better evaluate the necessity of a consultation or take action on their own. Almost all participants stressed the importance of personal encounters with the GP, especially in times of increased need for care. They stressed that online access could not replace personal contact but should be handled as an additional tool for healthcare: “If you really have some problem, you still want to be seen by the GP. I don’t think that’s going to lead to fewer appointments” (P6, male, 18–34 years); and “Access to your medical records, that has additional value. But that first step is still the personal contact” (P2, female, 50–65 years).

## Discussion

This qualitative study explored Dutch adults’ needs and expectations regarding online access to general practice medical records. Generated insights were grouped into three overlapping areas: prerequisites for getting online access, using online access, and the impact on interaction with healthcare providers. Across all three areas, participants identified several obstacles and needs to use online access. First, only a few participants had previous experience with online access to GP medical records and many participants had not even heard about the option yet. This can be explained by the facts that patients’ legal entitlement to online access in the Netherlands had been recently implemented (July 2020) and little publicity was given prior to the interviews. This obstacle of low levels of awareness has been found in various countries (Powell, 2017; Van Kasteren et al., 2017). Our study suggests that efforts to raise awareness about online access can already increase intended use, and that patients expect the GP practice to take up an active role in this. However, an umbrella review including research from various countries on healthcare providers’ attitudes towards online access shows they are concerned about anxious, overwhelmed and offended patients, liability and changes in workload (Antonio et al., 2020). These concerns might create resistance to introduce patients to online access and have to be explored and addressed to implement online access successfully.

Second, participants in this study were concerned that creating an account and using online access would be difficult for them, which is similar to findings from Lyles et al. (2020) that limited digital literacy and confidence of patients are among the most common barriers to online access use among various populations. As 36% of the Dutch population are considered as having inadequate or limited functional/cognitive health literacy skills (Rademakers and Heijmans, 2018), this obstacle deserves attention in the implementation process. Improvements of accessibility on portal level or additional trainings for vulnerable groups could address this issue (Forchuk et al., 2015). Positive effects of a support strategy for portal use have already been demonstrated (Ramsey et al., 2018), but additional research is needed on the needs of diverse patient groups, especially those with low digital literacy (Lyles et al., 2020).

Third, almost all participants were worried about the privacy and confidentiality of their data, which is a common concern observed in various populations (Lyles et al., 2020; Powell, 2017). Interestingly, participants of this study seemed willing to accept the perceived risk of security breach as most of them still intended to use online access. Although the worries might thus not decrease user rates, they can possibly interfere with intended effects such as increased patient satisfaction. Some privacy concerns mentioned in our interviews were based on misconceptions, for example, that alongside patient access other healthcare providers automatically have access to the data as well. Misconceptions might be resolvable with educational efforts. Additionally, privacy and security of data should be prioritised in the development of and education about online access.

Fourth, participants wanted to use online access to view and review their medical record and thought that thereby they would gain more knowledge and understanding about their health. However, they were also concerned that the medical language used in their record is incomprehensible for them. Furthermore, not understanding health data could lead to confusion, concerns and wrong interpretations which might even lead the patient to take unanticipated and potentially harmful actions. Due to this ambivalence, online access has been described as a double-edged sword (Baun et al., 2020; Lester et al., 2016), which can be either beneficial or harmful, depending on the patient’s comprehension of the data. The notion that medical records were initially only developed to be used (and therefore understood) by healthcare professionals makes it obvious that people without medical background might struggle with comprehending the presented health information (Beard et al., 2012). It is crucial to explore how documentation practices can be changed to serve the needs of both healthcare professionals and patients, and how patients with limited health literacy or insufficient knowledge can be best supported to interpret medical data correctly.

Overall, results of this study suggest that to address the obstacles of low awareness, low digital literacy, security concerns and complex language in medical records, implementation accompanying strategies are necessary. In line with research priorities listed by Lyles et al. (2020), we propose that future research should address the key role of the GP and staff in promoting and facilitating online access as well as the role of the government in supporting them.

By being able to access and understand their data, participants in this study expected to feel empowered and enabled to contribute more to conversations and decisions regarding their health(care). While these expectations support commonly envisioned effects of online access (Tapuria et al., 2021), systematic reviews conclude that to date, the actual influence on patient empowerment and decision-making remains under-investigated (Fraccaro et al., 2018; Ammenwerth et al., 2019). Research is needed to understand the complex process of how online access might improve health outcomes (Fraccaro et al., 2018). Our study indicates important needs and expectations that can be included in such investigations. Further, to understand how effects of online access relate to patient satisfaction, we recommend including measurement of patients’ evaluation of those effects in further investigations of the impact of online access to medical records.

### Limitations

When interpreting the results of this study, certain limitations have to be considered. First, due to the novelty of online access in the Netherlands, participants’ imaginability of online access and its potential effects might be limited. To account for this, we showed a brief video, explaining online access, at the beginning of the interview and got the impression and feedback that thereafter understanding was sufficient. Second, it should be noted that sampling bias might have led to participants of this study having a more positive attitude towards online access and imagination of its effects compared to people that did not participate. Overall, we believe that we were able to generate in-depth findings from a diverse group of participants pointing towards priorities for implementation strategies and future research.

## Conclusion

This study demonstrated that patients expected benefits from online access such as better overview, personal empowerment, and improved communication with their GP, while they were concerned about technological difficulties, data privacy, and complex medical language in their record. Organisational changes in general practice, for example, adjusted documentation practices, and implementation accompanying strategies, such as educational efforts, are needed to support patients to access, understand and use their medical record and to eventually achieve desired outcomes of increased patient participation and satisfaction.
